# YAP, ΔNp63, and β-Catenin Signaling Pathways Are Involved in the Modulation of Corneal Epithelial Stem Cell Phenotype Induced by Substrate Stiffness

**DOI:** 10.3390/cells8040347

**Published:** 2019-04-12

**Authors:** Ricardo M. Gouveia, Flora Vajda, Jason A. Wibowo, Francisco Figueiredo, Che J. Connon

**Affiliations:** Institute of Genetic Medicine, Newcastle University, International Centre for Life, Newcastle-upon-Tyne NE1 3BZ, UK; vajdaflo@gmail.com (F.V.); jasonowo.anthony@hotmail.com (J.A.W.); francisco.figueiredo@newcastle.ac.uk (F.F.)

**Keywords:** tissue stiffness, limbal epithelial stem cells, mechanotransduction, epithelial stratification, β-Catenin signaling, fibrosis

## Abstract

Recent studies have established that the phenotype of epithelial stem cells residing in the corneal periphery (the limbus) depends on this niche’s distinct biomechanical properties. However, the signaling pathways underlying this dependency are still poorly understood. To address this issue, we investigated the effect of substrate stiffness on the migration, proliferation, and molecular phenotype of human limbal epithelial stem cells (LESCs). Specifically, we demonstrated that cells grown on collagen-based substrates with limbus-like compliance showed higher proliferation and stratification and lower migration capabilities, as well as higher levels of pro-proliferative markers Ki67 and β-Catenin, and LESC markers ΔNp63, ABCG2, and CK15. In contrast, cells on stiffer substrates lost these stem/progenitor cell markers, but instead expressed the key mechanotransduction factor YAP, as well as elevated levels of BMP4, a promotor of cell differentiation known to be negatively regulated by Wnt/β-Catenin signaling. This data allowed us to propose a new model that integrates the various molecular pathways involved in LESC response to substrate stiffness. This model will potentially be a useful guide to future research on the mechanisms underlying LESC loss following fibrosis-causing injuries.

## 1. Introduction

The cornea is the outermost tissue of the eye, and its transparency is determinant for maintaining vision quality. On its anterior side, the cornea is covered by a nonkeratinized, stratified squamous epithelium that plays an essential role in the tissue’s function [1]. In particular, the corneal epithelium provides an important barrier for invading pathogens as well as to fluids, and thus represents an important regulator of tissue hydration, transparency, and homeostasis. This crucial role is in turn dependent on a balanced epithelial turn-over, with the older, more apical cells lost by debridement being consistently replaced by new cells originating from the tissue’s periphery, the limbus [2].

A considerable amount of evidence has established that, in the human cornea, the limbus is populated by residing epithelial stem/progenitor cells [2,3]. Moreover, numerous studies have shown that these limbal epithelial stem cells (LESCs) are responsible for epithelial self-renewal via a slow but continuous process of proliferation, followed by differentiation, and centripetal migration towards the central region of the cornea [4,5,6]. Several biochemical and biophysical factors have been suggested to play a critical role in regulating this behavior [7,8]. For example, the development of a differentiated epithelium has been found to depend on Wnt/β-Catenin-mediated expression of Bone Morphogenetic Protein 4 (BMP4) from stromal cells [9]. In addition, the limbus comprises a distinct (more compliant) biomechanical niche opposed to the relatively stiff corneal center [10,11,12], and that LESC maintenance is highly dependent on these softer mechanical properties [12,13,14]. Conversely, we recently demonstrated that the stiffening of the limbus matrix (e.g., due to chemical injury) promotes epithelial stem cell differentiation via mechanotransduction-dependent pathways [15], negatively impacting their renewal and ultimately leading to stem cell deficiency, corneal opacification, and vision loss.

These findings highlighted the importance of corneal tissue biomechanics for maintaining epithelial homeostasis [13]. This is relevant as it illustrates starkly the intimate interaction between the corneal epithelium and its underlying supporting stroma [8,11,16]. Therefore, in this study we further explored the impact of these interactions by testing the effect of tissue stiffness on epithelial stem cell migration, proliferation, and stratification in vitro, as well as on the expression of several master regulators associated with these processes. Our findings subsequently allowed us to propose a molecular model integrating probable signaling pathways involved in LESC mechanotransduction responses.

## 2. Materials and Methods

### 2.1. Primary Cell Isolation and Culture

**Figure 1:**
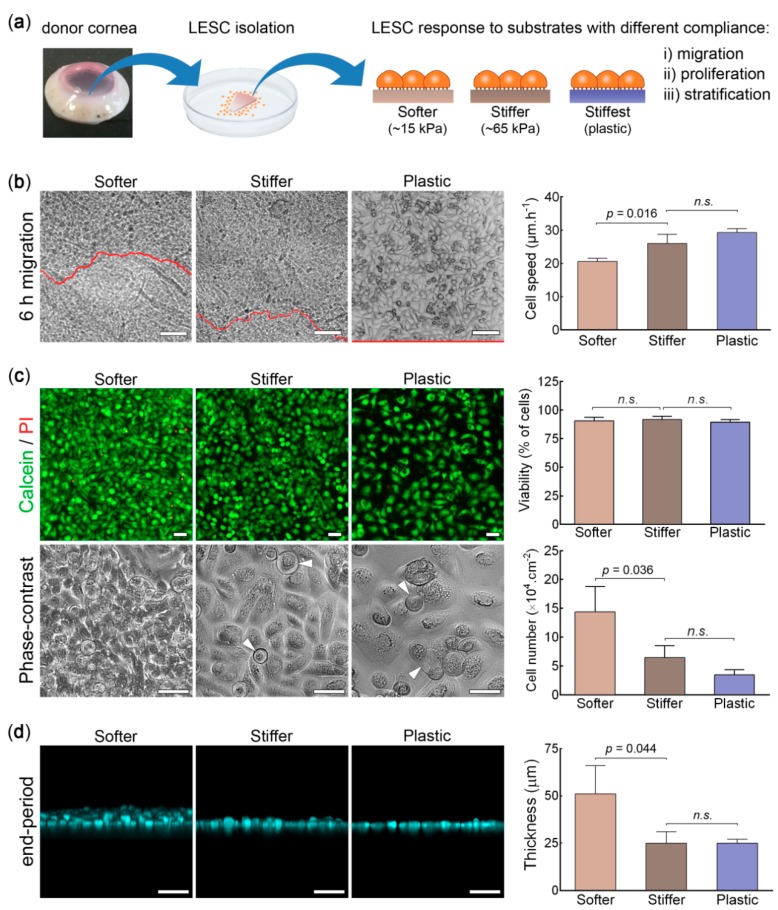
The effect of substrate compliance on human limbal epithelial stem cells (LESCs). (**a**) Experimental setup. Cells isolated and expanded from donor corneal explants were seeded onto collagen gel substrates with limbus-like compliance (~15 kPa) or stiffer (~65 kPa) [13]. Cell migration, proliferation, and stratification were analyzed in separate experiments, with normal tissue culture plastic used as infinite stiffness control. (**b**) Bright-field micrographs of LESCs migrating on substrates with different stiffness and quantification of corresponding cell speed (µm h^−1^). The red line represents the front of cell migration after 6 h. (**c**) Live/dead (green/red) double cell staining (upper) and phase-contrast micrographs (lower panel) of LESCs grown for 15 days on substrates with different stiffness, with corresponding quantification of cell viability and proliferation. Round, detaching cells are highlighted (arrowheads). (**d**) Effect of the different substrate compliance on LESC stratification. Cells grown for 15 days formed a continuous epithelium, which was then imaged in cross-section by confocal fluorescence microscopy after bisbenzimide staining, and analyzed for its thickness. All plotted data corresponds to the average ± s.d. from three independent experiments (n = 3; n.s., nonsignificant). Scale bars, b = 100 µm; c, d = 50 µm.

Limbal epithelial stem cells (LESCs) were isolated from explants of cadaverous human corneal rings remaining from donor tissue (six donors aged 65–76 years old; male–female donor ratio of 1:1; no prior history of corneal diseases or ocular trauma) following removal of the central 7 mm for keratectomy, in accordance with the Newcastle University and Newcastle-upon-Tyne Hospital Trust Research Ethics Committees’ guidelines [17]. Briefly, human corneal rings including the limbus region were dissected into quarters, with remaining scleral tissue removed, plated in 6-well plates, and subsequently incubated in 4 mL of CnT-07 medium (CellNTec, Switzerland) for up to ten days at 37 °C and 5% CO_2_, with culture medium change every two days, to allow LESC attachment and expansion (Figure 1a). LESC monolayers reaching 70–80% confluence were then passaged using Accutase cell detachment solution (Thermo Fisher Scientific, Waltham, MA, USA) for 10 min at 37 °C, followed by centrifugation, resuspension, and replating for further expansion on T25 flasks at 3 × 10^4^ cells.cm^−2^ up to passage 3 or on collagen gels at 5 × 10^4^ cells.cm^−2^ to test the effect of substrate stiffness on their phenotype (Figure 1a).

### 2.2. Collagen Gel Fabrication

High-density collagen gels were prepared by mixing rat tail collagen type I (First Link, Birmingham, UK), 10× MEM (Thermo Fisher Scientific), and 1M NaOH (Merck, Darmstadt, Germany) solutions at a 8:1:1 volume ratio in a 50 mL falcon tube, followed by centrifugation at 1000× *g* at 4 °C for 5 min to remove air bubbles. This liquid gel mix was then distributed into 1 mL aliquots into 24-well tissue culture plates and incubated for 30 min at 37 °C to solidify. Subsequently, gels were plastic-compressed by applying RAFT absorbers (Lonza, Basel, Switzerland) to their top surface for 10 min. The resulting ~150-µm-thick gels were then treated with a collagenase type I enzyme (Thermo Fisher Scientific) in order to soften them and obtain a limbus-like compliance (~15 kPa). Briefly, collagenase dissolved in phosphate buffer saline (PBS; Merck) at 25 mg L^−1^, and 1 mL of this solution was then used to treat the plastic-compressed gels for 1 h at 37 °C. Gels treated with PBS alone were used as stiffer (~65 kPa) substrate counterparts [13]. Following these treatments, gels were washed thrice with an excess of PBS and then incubated overnight at 37 °C with 0.5 mL of fetal bovine serum (FBS; BioSera, Nuaille, France) to neutralize any remaining enzyme. Finally, all gels were treated with 1 µg L^−1^ laminin solution (Thermo Fisher Scientific) for 1 h and at 37 °C to create a surface coating that promotes LESC adhesion.

### 2.3. Cell Migration Assay

Collagen gels treated with collagenase (softer) or PBS (stiffer) were seeded with 1 × 10^5^ LESCs in 1 mL of CnT-07 medium, and incubated for 24 h at 37 °C with the substrate held at an initial 45° tilt to ensure cells only attached to the lower half surface of the gel, thus forming a defined cell boundary with the upper half above the air–liquid interface. Subsequently, cultures were washed thrice with PBS to remove unattached cells, and incubated at 37 °C for 24 h fully submerged in CnT-07 medium. Cells were imaged every 10 min by time-lapse bright-field microscopy using a Lumascope 500 inverted microscope (Etaluma, San Diego, CA, USA) to track their migration. Micrographs were binarized using the ImageJ v1.7 software to better determine the position of individual cells in each image frame. Cell speed (µm h^−1^) was evaluated by determining the position of 100 individual cells during the initial 6 h of migration, and tracing total distance covered by moving cells and their migration front using the standard parameters of the wrMTrck plugin for ImageJ v1.7. Data was expressed as the average ± standard deviation (s.d.) from three independent experiments (n = 3), each performed with cells from a different donor.

### 2.4. Cell Viability and Proliferation Assay

Collagen gels treated with collagenase (softer) or PBS (stiffer) were transferred into Transwell culture inserts (Corning, Corning, NY, USA) and seeded with 1 × 10^5^ LESCs suspended in 1 mL of CnT-07 medium. Plastic coverslips (Agar Scientific, Stansted, UK) similarly coated with 1 µg L^−1^ laminin were used as infinite stiffness control substrates (Figure 1a). Cells were allowed to attach overnight at 37 °C, and confirmed to cover all surfaces uniformly the following day by phase-contrast microscopy. LESCs on the softer, stiffer, and plastic substrates were subsequently cultured for 15 days, with medium replacement every 2 days, and then analyzed for their viability using Live/Dead double staining assay (Merck), as previously described [18], and for proliferation using the AlamarBlue assay. Briefly, gels were incubated with resazurin reagent (Merck) diluted 1:10 in fresh culture medium and incubated for 4 h at 37 °C, after which 100 µL of culture supernatants were sampled (in triplicate) for fluorescence emission analysis at 590 nm using a Fluoroskan Ascent plate fluorometer (Thermo Fisher Scientific). Cell number was calculated by interpolation using a standard curve for fluorescence values of 1, 5, 10, 20, and 50 × 10^4^ cells, with values corresponding to the average ± s.d. from three independent experiments (n = 3), each performed with cells from a different donor.

### 2.5. Immunohistochemistry

Cells grown on either softer and stiffer collagen gels or plastic coverslips were washed in PBS, fixed in 4% paraformaldehyde for 20 min, washed with excess PBS, incubated with blocking solution comprising 5% bovine serum albumin (First Link) and 0.1% Triton X-100 (Merck) in PBS for 1 h, and then incubated overnight at 4 °C with the following primary antibodies diluted 1:500 in blocking buffer; mouse β-Catenin and CK15, rabbit BMP4 and Ki67, and rat ABCG2 (ab124715, ab52816, ab11350, ab15580, and ab24115, respectively; Abcam, Cambridge, UK); rabbit ΔNp63, goat YAP, and mouse CK3 (sc-8343, sc-17141, and sc-80000, respectively; Santa Cruz Biotechnology, Dallas, TX, USA). Cells were then washed vigorously four times for 15 min in PBS containing 0.1% Triton X-100, and incubated for 2 h at room temperature and in the dark with corresponding secondary antibodies and bisbenzimide nuclear stain (Hoechst 33342; Thermo Fisher Scientific) diluted 1:1000 and 1:5000 in blocking buffer, respectively. After another four 15-min washes, both gels and plastic coverslips were immersed in antifade medium (Vector Laboratories, Peterborough, UK), mounted on glass slides, and imaged by confocal fluorescence microscopy using a Nikon A1, with 1 µm-thick optical sections and constant illumination and capture parameters. Data were analyzed using the NIS-Elements and the ImageJ v.1.7 software suite, with expression quantified by evaluating pixel intensity for each independent channel or by calculating the percentage of cells with nuclear ΔNp63, β-Catenin, Ki67, or YAP and normalizing it (in percentage) against the control (corresponding plastic substrates). Representative images were taken from each independent sample, for all three experiments (n = 3).

### 2.6. Statistics

Data was analyzed using a one-way (epithelial cell number, viability, migration, and stratification assays) or two-way analysis of variance (ANOVA) (marker expression) followed by Bonferroni’s multiple comparison *post hoc* test. Significance between groups was established for *p* < 0.05, 0.01, and 0.001, with a 95% confidence interval. For all assays, error bars represented the s.d. of the mean, analyzed *a priori* for homogeneity of variance.

## 3. Results

To evaluate the effect of substrate compliance on LESC migration, cells were seeded onto half the area of three distinct culture surfaces (i.e., softer and stiffer collagen gels or tissue culture plastic; Figure 1a), and then imaged for 6 h by time-lapse microscopy (Figure 1b). In all conditions, LESCs migrated collectively from the original seeding site into the contiguous, cell-free surface, moving as a continuous sheet (Figure 1b). Interestingly, cells on limbus-like (softer) collagen substrates moved slower compared to cultures on stiffer substrates, as evidenced by their fronts of migration (Figure 1b, red line). Specifically, LESCs on softer substrates showed to move at an average 20 ± 2 µm h^−1^, a significantly lower migration rate compared to the 26 ± 2 µm h^−1^ from cells on stiffer collagen substrates (*p* = 0.016). This latter condition supported a similar migration rate to that of cell on plastic (29 ± 2 µm h^−1^) (Figure 1b).

Substrate stiffness also affected cell proliferation and stratification. LESCs seeded and grown evenly on softer substrates proliferated for the entire 15 days period of culture, remaining highly viable (Figure 1c) and showing evident signs of stratification (Figure 1d). In contrast, cells grown on both stiffer collagen gels and plastic maintained a single monolayer, with any of the few stratifying cells rapidly becoming round and detaching from the basal cell sheet (Figure 1c, arrowheads). Consequently, and despite the comparable viability levels (Figure 1c; upper panel), this corresponded to softer substrates presenting a significantly higher cell number compared with stiffer ones (*p* = 0.036) (Figure 1c; lower panel). Moreover, the epithelium formed on softer collagen gels was significantly thicker than that on stiffer substrates (*p* = 0.044) (Figure 1d). Specifically, the additional one or two suprabasal epithelium layers formed on softer substrates showed an average thickness of 51 ± 15 µm, whereas single-layer epithelia on stiffer collagen substrates and tissue culture plastic presented approximately half that thickness (25 ± 6 and 25 ± 2 µm, respectively, Figure 1d). The lower epithelium thickness was both due to the lower cell number and the flatter, more stretched morphology of cells grown on stiffer collagen and plastic substrates (Figure 1c).

**Figure 2:**
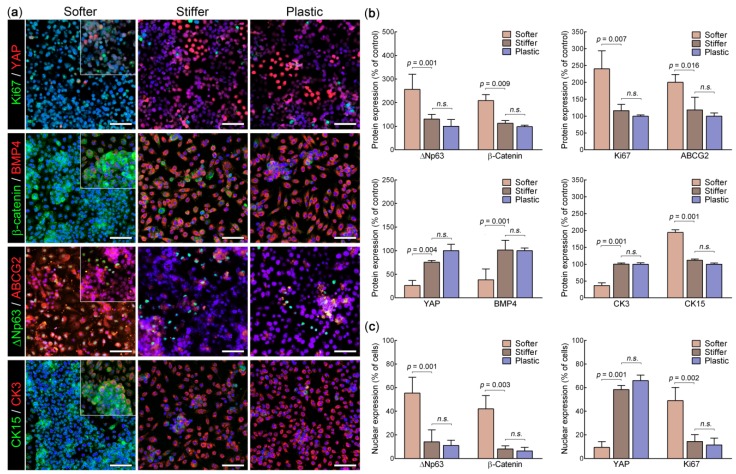
The effect of substrate stiffness on human limbal epithelial stem cell molecular phenotype. (**a**) LESCs grown for 15 days on collagen gel substrates with limbus-like compliance (softer) or stiffer mechanical properties, as well as on normal tissue culture plastic (control) were imaged by confocal immunofluorescence microscopy for their expression of both corneal epithelial stem/progenitor cell markers (ΔNp63, ABCG2, and CK15), proliferation markers (Ki67 and β-Catenin), and differentiation markers (YAP, BMP4, and CK3). Cell nuclei were detected using bisbenzimide (blue staining). Insets correspond to suprabasal cell layers from softer substrate cultures. Images correspond to the top view of confocal *z*-stack tridimensional reconstruction. Scale bars, 100 µm. (**b**) Marker expression was analyzed from ten random areas in each experiment, with average ± s.d. expression calculated from three independent experiments (n = 3; n.s., nonsignificant) after normalization to the control (average expression on plastic). (**c**) The nuclear localization of ΔNp63, β-Catenin, Ki67, and YAP was analyzed using the same micrographs, and calculated as the average ± s.d. percentage of total cells.

The effect of substrate stiffness on epithelial cell phenotype was further examined by evaluating the expression of specific corneal epithelial stem/progenitor cell markers, and of markers of cell proliferation and corneal epithelial cell differentiation. Cells grown for 15 days on softer collagen gel substrates expressed higher levels of LESC-characteristic markers ΔNp63, ABCG2, and CK15 [3,19,20], as well as the pro-proliferation factors Ki67 and β-Catenin [21,22] compared with those on stiffer gels (Figure 2a). These differences corresponded to significant 2-, 1.7-, and 1.7-fold increases in signal intensity for ΔNp63, ABCG2, and CK15, respectively (*p* = 0.001, 0.016, and 0.001, respectively), and 2- and 1.9-fold increases for Ki67 and β-Catenin, respectively (*p* = 0.007 and 0.009, respectively) (Figure 2b). Cells grown on softer substrates also showed a significantly lower expression of differentiation markers compared with those on stiffer (or plastic) substrates, namely CK3 (a marker of differentiated corneal epithelial cells [2]), BMP4 (a key cell differentiation factor [9,23]), and YAP, an important mechanotransduction regulator (*p* = 0.001, 0.001, and 0.004, respectively) (Figure 2b). Furthermore, marker activity followed their expression levels, with cultures grown on softer collagen gel substrates displaying significantly higher percentage of cells with nuclear (active) ΔNp63, Ki67, and β-Catenin (*p* = 0.001, 0.002, and 0.003, respectively) and significantly lower percentage of cells with nuclear YAP (*p* = 0.001) (Figure 2c). These differences were particularly evident in LESCs forming the basal layer of the stratified epithelium, as a few isolated cells from the suprabasal layers presented an expression profile closer to cells on stiffer substrates (Figure 2a; insets).

These results indicated that, in the specific conditions of this in vitro setting, the phenotype of human corneal epithelial cells was highly dependent on the mechanical properties of their substrate, with tissue compliance promoting cell proliferation, stratification, and the expression of LESC-characteristic markers, and stiffer substrates promoting corneal epithelial cell migration and differentiation. Interestingly, no significant differences in marker expression profile were observed between cells on stiffer collagen gel and plastic substrates (Figure 2), indicating that LESCs are particularly responsive to relatively soft environments.

## 4. Discussion

Previous studies have established that the limbus represents as softer biomechanical niche compared with the central cornea [10,11], suggesting that such compliance is sensed by, and translated within, stem cells [13,24]. Moreover, the softer mechanical properties of the limbus have been hypothesized to promote a number of molecular pathways important for LESC maintenance, whereas stiffer conditions have been associated with increased cell activation and differentiation [13,15]. In this study, the use of collagen-based substrates that emulate the softness of the corneal limbus indicated that, at least in vitro, YAP, ΔNp63, and β-Catenin represent important molecular elements underlying the mechanotransduction responses from human LESCs. In particular, we demonstrated that the limbus-like soft substrates promote cell proliferation and stratification without affecting cell survival. Correspondingly, LESCs grown on these softer substrates showed higher expression of LESC-characteristic markers while presenting low YAP expression and activation. In contrast, stiffer substrates inhibited LESC proliferation and stratification, promoting cell differentiation and enhancing YAP expression and activation. YAP is a well-known molecular regulator of cell mechanotransduction, with environmental stiffness often leading to its increased expression, activation, and nuclear translocation [25]. Together with its coeffector TAZ, YAP interacts with TEA domain transcription factors (TEADs) and promotes the expression of focal adhesion genes [26,27]. Moreover, YAP activation has been associated with both direct and indirect downregulation of ΔNp63 [27], Wnt/β-Catenin [28], and ABCG2 expression [29].

Furthermore, our results suggested that the stiffness-induced behavior of LESCs may be stimulated by BMP4, an important pro-differentiation growth factor known to be tightly interconnected with mechanotransduction signaling during development [9], regeneration [30], and homeostasis [23,31] of several types of epithelia. Previous studies have shown that BMP4 has a crucial role in cell differentiation and morphogenesis in the eye. In mouse corneas, the BMP4 released by stromal cells has been suggested to function as a key promotor of corneal epithelial differentiation and stratification via regulation of ΔNp63 activity [9,32]. In this system, β-Catenin works as negative regulator of BMP4 expression in stromal cells, thus controlling a highly-regulated, BMP4-mediated stoma–epithelium cross-talk. Moreover, BMP4 inhibits the long-term expansion of ΔNp63-positive airway epithelial cells in vitro via modulation of SMAD signaling [33].

**Figure 3:**
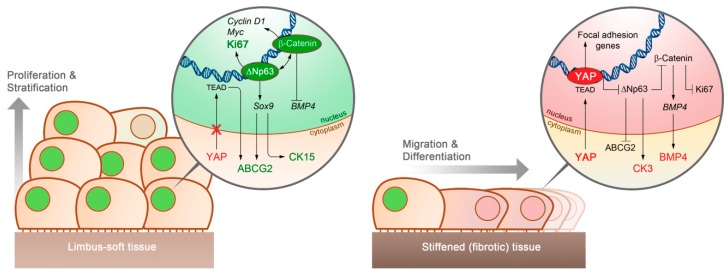
A depiction of how substrate stiffness can affect the behavior of corneal epithelial stem cells via mechanotransduction. In our proposed model, cells grown on limbus-like soft tissues (left panel) show impaired YAP activation and nuclear translocation, which then allows the major cell proliferation regulators β-Catenin and Ki67 and the LESC markers ABCG2 and CK15 to be highly expressed via ΔNp63 activation. Nuclear β-Catenin also contributes to stem cell maintenance by preventing BMP4-induced cell differentiation. However, substrate stiffening, e.g., due to fibrosis in the limbus (right panel), induces YAP nuclear translocation, which in turn suppresses Wnt/β-Catenin both directly and indirectly via inhibition of ΔNp63 signaling. The YAP-mediated inhibition of ΔNp63 and β-Catenin not only results in the loss of pro-proliferative factors (e.g., Ki67) and LESC markers (ABCG2 and CK15), but also promotes the expression of the cell differentiation factor BMP4 and markers of differentiated corneal epithelial cells (e.g., CK3). The direct upregulation of focal adhesion genes by an active nuclear YAP also explains the higher migration rate of epithelial cells on stiffer substrates.

This data allowed us to propose a new mechanistic model to explain how substrate stiffness is able to regulate LESCs behavior and phenotype (Figure 3). In this model, the softer properties of the limbus promote the inactivation of YAP and the maintenance of high expression levels of ΔNp63, β-Catenin [34], and ABCG2 [35] (Figure 3, left panel). The nucleus-expressed ΔNp63 also reinforces YAP inactivation [36] and Wnt/β-Catenin signaling [37], while promoting the expression other stem/progenitor cell markers (e.g., ABCG2 and CK15) via Sox9 activation [38]. Subsequently, β-Catenin can act as an inducer of pro-proliferation factors (e.g., Ki67, Cyclin D1, and Myc) [22,39,40] as well as an inhibitor of BMP4 expression [9]. Together, these pathways would promote LESC proliferation and stratification, with stem cell phenotype being maintained via inhibition of BMP4-mediated cell differentiation. However, as LESCs stratify, the influence of the soft substrate becomes less pronounced, leading to a progressive differentiation of suprabasal cells (Figure 3, left panel), possibly via YAP activation.

Similarly, in cases where a stiffening of the limbus occurs, e.g., due to tissue fibrosis or chemical injury, the resulting YAP activation and nuclear translocation initiate a signaling cascade that alters LESC behavior (Figure 3, right panel). In particular, the YAP-related promotion of focal adhesions both at transcriptional and protein levels [26] allows cells to become more migratory, an important feature in wound healing responses. In addition, stiffness-induced YAP activation can inhibit ABCG2 expression [29,35] and Wnt/β-Catenin signaling both directly [34] and indirectly via suppression of ΔNp63 [41], consequently leading to increased BMP4 [42] and CK3 expression [38] and, ultimately, cell differentiation (Figure 3, right panel). This theory was supported by previous studies showing that chronic inflammation in the corneal limbus can lead to increased stromal stiffness, YAP activation, and β-Catenin-mediated deficiencies in LESC maintenance [43]. These conclusions are also in line with current knowledge on mechanotransduction in adult stem cells, particularly in other types of epithelia [44].

Overall, this integrated model establishes feasible links between YAP-dependent mechanotransduction, ΔNp63 and Wnt/β-Catenin signaling, and consequent BMP4-mediated cell differentiation. In this perspective, this study creates a new basis for future studies aiming at understanding in detail the molecular pathways controlling corneal epithelial cell in both health and disease states.
